# Rapid and High-Throughput Reverse Transcriptase Quantitative PCR (RT-qPCR) Assay for Identification and Differentiation between SARS-CoV-2 Variants B.1.1.7 and B.1.351

**DOI:** 10.1128/Spectrum.00506-21

**Published:** 2021-10-06

**Authors:** Oran Erster, Ella Mendelson, Virginia Levy, Areej Kabat, Batya Mannasse, Hadar Asraf, Roberto Azar, Yaniv Ali, Rachel Shirazi, Efrat Bucris, Dana Bar-Ilan, Orna Mor, Michal Mandelboim, Danit Sofer, Shai Fleishon, Neta S. Zuckerman

**Affiliations:** a Central Virology Laboratory, Public Health Services, Ministry of Health, Chaim Sheba Medical Center, Ramat Gan, Israel; b School of Public Health, Sackler Faculty of Medicine, Tel-Aviv University, Tel-Aviv, Israel; University of Mississippi Medical Center

**Keywords:** SARS-CoV-2, RT-qPCR, variant B.1.1.7, variant B.1.351, rapid diagnosis, differential PCR, Sanger sequencing, Illumina sequencing, Alpha variant, Beta variant, real-time PCR, SC-2 variants, molecular diagnostics, molecular virology

## Abstract

Emerging SARS-CoV-2 (SC-2) variants with increased infectivity and vaccine resistance are of major concern. Rapid identification of such variants is important for the public health decision making and to provide valuable data for epidemiological and policy decision making. We developed a multiplex reverse transcriptase quantitative PCR (RT-qPCR) assay that can specifically identify and differentiate between the emerging B.1.1.7 and B.1.351 SC-2 variants. In a single assay, we combined four reactions—one that detects SC-2 RNA independently of the strain, one that detects the D3L mutation, which is specific to variant B.1.1.7, one that detects the 242 to 244 deletion, which is specific to variant B.1.351, and the fourth reaction, which identifies the human RNAseP gene, serving as an endogenous control for RNA extraction integrity. We show that the strain-specific reactions target mutations that are strongly associated with the target variants and not with other major known variants. The assay’s specificity was tested against a panel of respiratory pathogens (*n* = 16), showing high specificity toward SC-2 RNA. The assay’s sensitivity was assessed using both *in vitro* transcribed RNA and clinical samples and was determined to be between 20 and 40 viral RNA copies per reaction. The assay performance was corroborated with Sanger and whole-genome sequencing, showing complete agreement with the sequencing results. The new assay is currently implemented in the routine diagnostic work at the Central Virology Laboratory, and may be used in other laboratories to facilitate the diagnosis of these major worldwide-circulating SC-2 variants.

**IMPORTANCE** This study describes the design and utilization of a multiplex reverse transcriptase quantitative PCR (RT-qPCR) to identify SARS-COV-2 (SC2) RNA in general and, specifically, to detect whether it is of lineage B.1.1.7 or B.1.351. Implementation of this method in diagnostic and research laboratories worldwide may help the efforts to contain the COVID-19 pandemic. The method can be easily scaled up and be used in high-throughput laboratories, as well as small ones. In addition to immediate help in diagnostic efforts, this method may also help in epidemiological studies focused on the spread of emerging SC-2 lineages.

## INTRODUCTION

The recent emergence of new SARS-COV-2 (SC-2) variants of concern (VOC) B.1.1.7 in the United Kingdom and B.1.351 in South Africa, both characterized by increased transmissibility and potential vaccine resistance (1–4), prompted dedicated surveillance by Israel’s Central Virology Laboratory to monitor their incursion into Israel.

Since the genome of variant B.1.1.7 contains 23 unique mutations, and that of variant B.1.351 contains 18 unique mutations, compared with the original Wuhan strain, it is practically impossible to detect all of them in one quantitative PCR (qPCR) assay. Thus far, the Thermo Fisher SC-2 detection kit (CAT CCU002) has been utilized to identify suspected B.1.1.7 samples (3). One of the reactions in this kit is directed to the viral spike gene and is negative when the template sequence contains the 69 to 70 deletion, which is one of the B.1.1.7 variant mutations. However, this deletion was also detected independently, is not unique to the B.1.1.7 variant, and can therefore often be misleading. Moreover, the absence of the S reaction in this assay may result from inhibition of this reaction and therefore may not necessarily indicate the presence of the deletion. Variant B.1.1.7 was first reported in the United Kingdom on September 2020 and by December 2020 became the dominant strain in the country (2). Its increased infectivity led to its rapid spread, with severe consequences on public health and the global economy (4). Likewise, from its first detection in Israel on December 23, this variant now comprises over 90% of the positive cases (O. Erster and N. Zuckerman, unpublished data).

An additional SC-2 VOC is the B.1.351 variant, which contains both B.1.351-unique mutations and mutations that are present in other notable VOC, such as the SGF deletion in the *nsp6* gene and the N501Y substitution in the spike gene that is identified also in B1.1.7 (5). Like variant B.1.1.7, this variant was found to be more infectious than the wild-type (WT) strain. *In vitro* studies also show that it has increased resistance to serum of recovered and vaccinated patients, thereby posing a serious threat to the efficacy of current vaccination campaigns (6–8).

The emergence of these two variants, as well as other recently emerging SC-2 strains, necessitates constant improvement of the diagnostic tools used to combat the COVID-19 pandemic. In addition to performing rapid, sensitive, and specific detection of the viral RNA, diagnostic tests are now required to differentiate between circulating strains to provide valuable epidemiological data for policy decision making. The commercial assays developed recently, (http://www.kogene.co.kr/eng/sub/product/covid-19.asp) detect specific key mutations. However, recent studies showed that in spite of sharing such mutations, variants might differ in their antibody resistance capacity, prompting development of variant-specific assays (5, 9). These findings highlight the need to determine the identity of circulating strains, not only specific mutations. By designing a multiplex PCR assay that positively detects the presence of unique mutations that are strongly associated with variants B.1.1.7 and B.1.351, rapid, economical, high-throughput screening can be performed, enabling robust and specific identification of these variants in SC-2-positive clinical samples.

In this report, we describe a differential RT-qPCR assay that detects the presence of mutations strongly associated with variants B.1.1.7 and B.1.351. We demonstrate that implementation of this novel multiplex assay allows a sensitive and specific detection of SC-2 RNA, with the advantage of variant differentiation in a single assay.

## RESULTS

### Development of differential COV19 VOC RT-qPCR.

Analysis of mutations characteristic of variants B.1.1.7 and B.1.351 showed that some of them were present in both, such as the SGF deletion in *nsp6* (positions 11285 to 11294 in sequence NC_045512) or the N501Y substitution in the spike protein gene (nucleotide position 23094 in sequence NC_045512). Others, such as the 69 to 70 deletion and N501Y substitution, developed independently and were detected in samples not classified as variant B.1.1.7 (see Fig. S1 in the supplemental material). On the other hand, the N gene D3L mutation is strongly associated with variant B.1.1.7 and is not associated with other currently dominant variants. Likewise, the D215G mutation and the S gene 242 to 244 deletion are strongly associated with variant B.1.351 and have not been identified in other variants thus far (Fig. S2.). Therefore, these two regions were selected for the multiplex reaction design.

### Design of the variant B.1.1.7-specific reaction.

**FIG 1:**
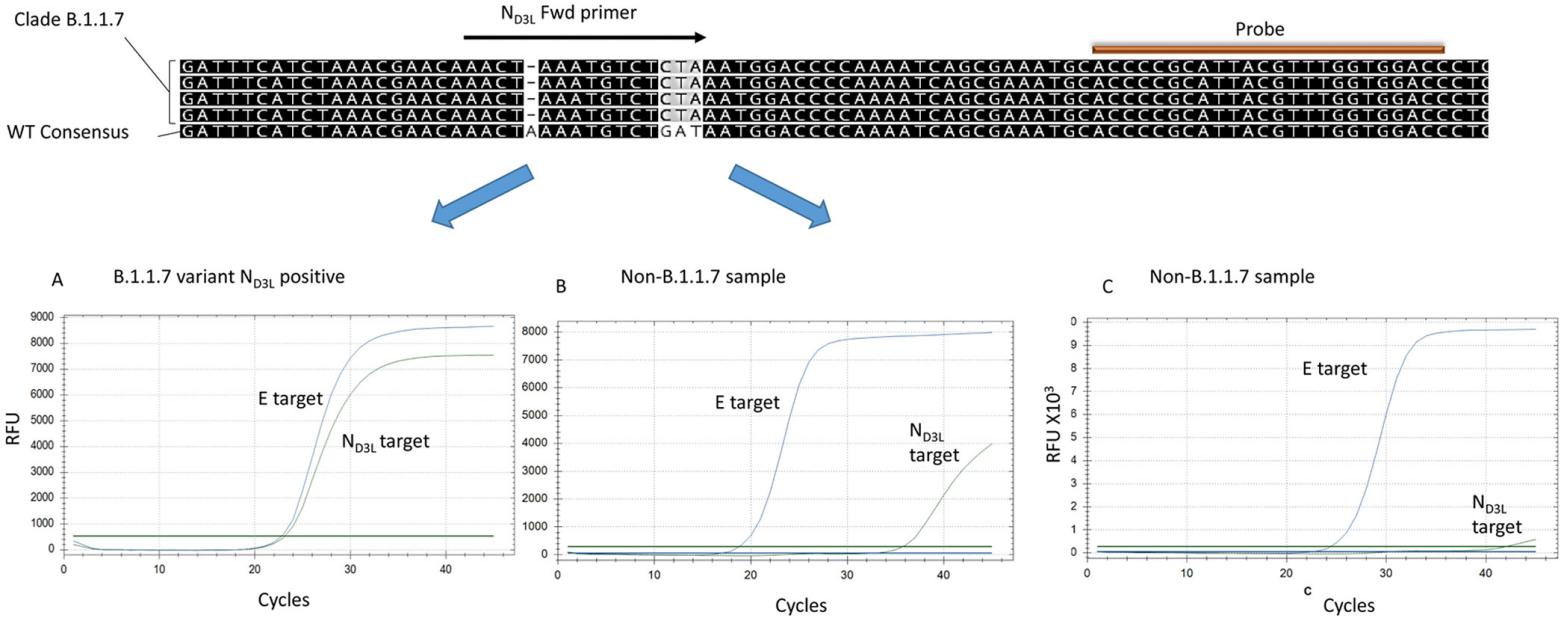
Design of variant B.1.1.7-specific reaction. A specific primer was designed based on the single nucleotide insertion and the codon substitution in the 5′p of the SC-2 N gene, as shown in the sequence alignment. The specific N_D3L_ reaction was combined with an inclusive E-sarbeco reaction (10) that detects all SC-2 variants. (A) The presence of the B.1.1.7 RNA in the assay results in two clear amplification curves. (B and C) A sample containing a non-B.1.1.7 variant sequence results in a faint amplification, with a *C_q_* difference larger than eight between the E and the N curves (B) or a negative signal for the N reaction (C).

**FIG 2:**
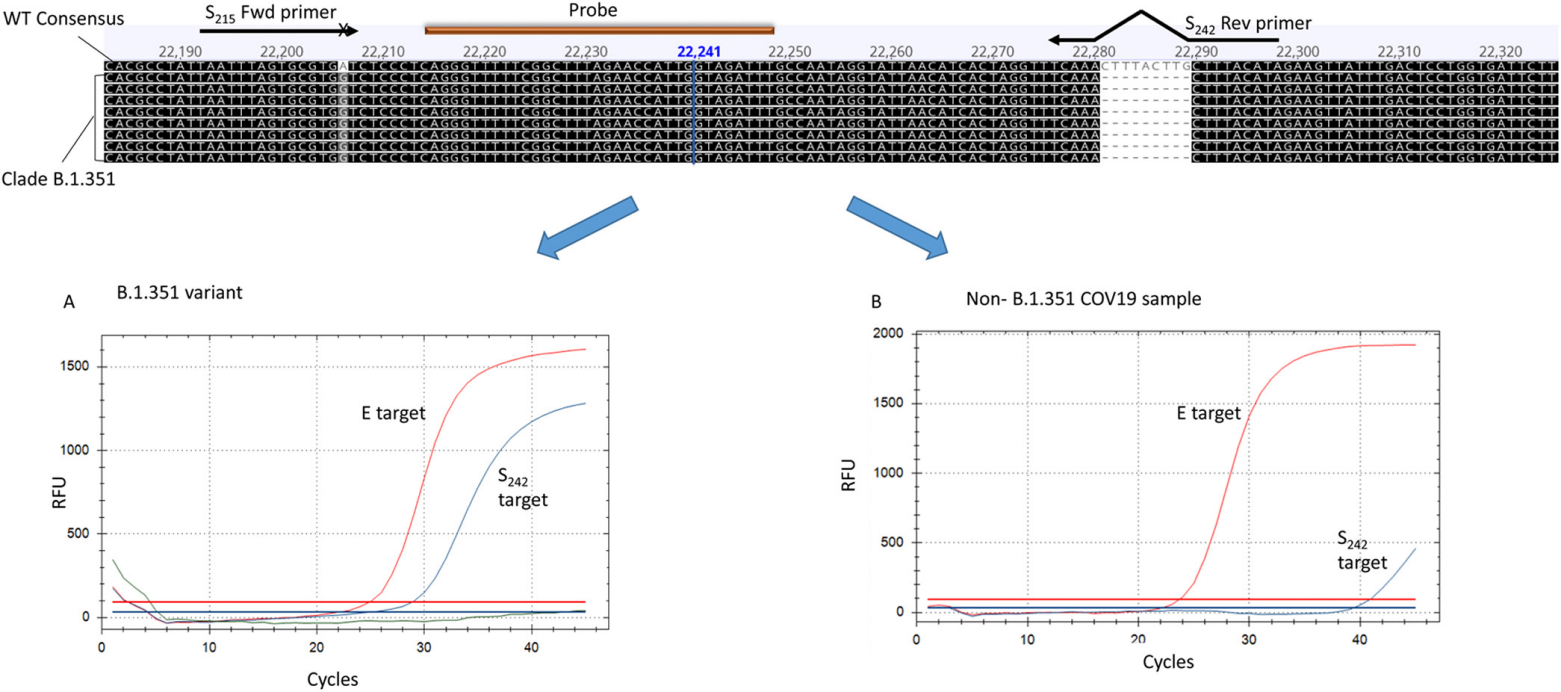
Design of variant B.1.351-specific reaction. Two primers were designed to specifically complement the sequences containing the D215G mutation and the 242 to 244 deletion. (A and B) The resulting reaction gives a clear signal of the S_242_ reaction with sample belonging to clade B.1.351 (A) and a negative, or a faint signal with a non-B.1.351 sample (B). The inclusive E-sarbeco reaction detects the presence of both B.1.351 and non-B.1.351 samples.

The 5′p region of the variant B.1.1.7 SC-2 N gene contains a complete codon substitution, translated into D3L amino acid substitution. Additionally, there is a single “A” deletion in this region, 5 bases upstream of the N gene start codon (Fig. 1). A selective primer was designed accordingly, to specifically detect the mutated variant. The reverse primer and the probe were based on the CDC N1 reaction (https://www.cdc.gov/coronavirus/2019-ncov/lab/rt-pcr-panel-primer-probes.html), as detailed in Table 1. The B.1.1.7-specific reaction was combined with an inclusive reaction based on the E-sarbeco qPCR described by Corman et al. (10), with minor modifications (Table 1). This reaction was designed to detect all known CoV19 clades, thereby serving as an indicator for the presence of SC-2 RNA, regardless of the variant type. The combined assay was then tested using sequence-verified samples of the Wuhan clade SC-2 (WT SC-2) and a sequenced lineage B.1.1.7 sample (Fig. 1). The Wuhan clade samples were either negative for the N reaction or gave a very weak signal, approximately 10 to 15 cycles apart from the E reaction signal (Fig. 1 and 2). This reaction was termed N gene D3L reaction—N_D3L_.

**TABLE 1:** Primers and probes used in this study for the multiplex PCR

Targeted region	Name	Sequence 5′→3′ and modifications	Reference or source
SC-2 E gene	E F1b	GTTAATAGCGTACTTCTTTTTCTTGC ^*a*^	10
SC-2 E gene	E-sarbeco R2	ATATTGCAGCAGTACGCACACA	10
SC-2 E gene	E-sarbeco probe	TXRed-ACACTAGCCATCCTTACTGCGCTTCG-BHQ2	10
N_D3L_	21257VOC Fwd	TAAACGAACAAACTAAATGTCTCTA	This study
N_D3L_	CDC N1 Rev	TCTGGTTACTGCCAGTTGAATCTG	CDC^*b*^
N_D3L_	CDC N1 probe	HEX-ACCCCGCATTACGTTTGGTGGACC-BHQ1	CDC^*b*^
S_D215G_ and S_242Del_	22201B SA Fwd	CGCCTATTATTTTAGTGCGTGG	This study
S_D215G_ and S_242Del_	22238 SA Rev	CAAATAACTTCTATGTAAAGTTTGAAAC	This study
S_D215G_ and S_242Del_	22230_probe	6-FAM-CAGGGTTTTTCGGCTTTAGAACCATTGG-BHQ1	This study
RNASE P gene	RNASE P Fwd	AGA TTT GGA CCT GCG AGC G	CDC^*b*^
RNASE P gene	RNASE P Rev	GAG CGG CTG TCT CCA CAA GT	CDC^*b*^
RNASE P gene	RNASE P probe	Cy5-TTC TGA CCT GAA GGC TCT GCG CG-BHQ2	CDC^*b*^

a The original sequence was modified to improve compatibility in the multiplex reaction.

b https://www.cdc.gov/coronavirus/2019-ncov/lab/rt-pcr-panel-primer-probes.html.

### Design of the variant B.1.351-specific reaction.

**FIG 3:**
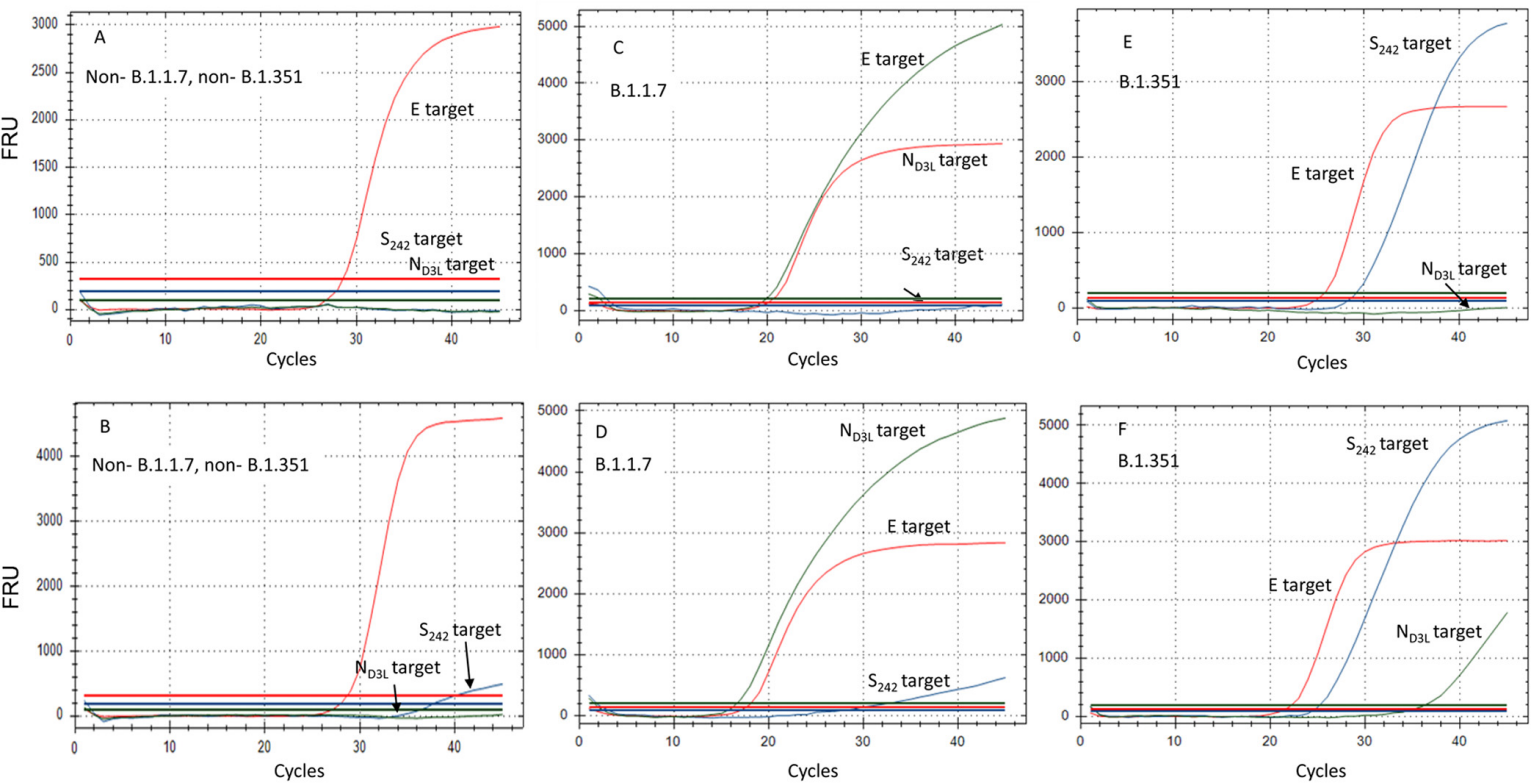
Amplification curve types of the multiplex reaction. The different outcomes of the multiplex assay are shown. (A) Non-B.1.1.7, non-B.1.351 sample, with no background signal. (B) Non-B.1.1.7, non-B.1.351 sample with S_242_ reaction background signal. (C) Variant B.1.1.7 sample with no background signal. (D) Variant B.1.1.7 sample with S_242_ background signal. (E) Variant B.1.351 sample with no background signal. (F) Variant B.1.351 sample with N_D3L_ background signal.

This variant contains two unique mutations in the spike gene, D215G and a deletion at amino acid position 242 (nucleotide positions 22281 and 22289 in sequence NC_045512). A specific reaction was developed, based on these mutations, which detects samples of lineage B.1.351, as described. The forward primer was designed to anneal to the substituted nucleotide at its 3′p end. In order to increase the primer selectivity, a mismatched base was inserted 10 bp downstream to the 5′p end. The reverse primer was designed to complement the region of the 242 to 244 deletion (Fig. 3). This reaction resulted in a negative, or very weak, signal when testing WT samples and a clear signal when testing samples previously sequenced and identified as belonging to the B.1.351 clade (Fig. 2 and 3). This reaction was then termed the S_242_ reaction. The virus of the first B.1.351 patient in Israel was cultured at the Israel Central Virology Laboratory (CVL), and the culture-derived RNA was used to calibrate the reaction. Using this assay, 64 samples were identified as variant B.1.351 and were confirmed by sequencing to contain the D215G and S_242_ mutations (Table S2).

### Generation and evaluation of the multiplex SC-2 VOC assay.

The two reactions were combined with the E target reaction and a reaction targeting the RNase P gene (https://www.cdc.gov/coronavirus/2019-ncov/lab/rt-pcr-panel-primer-probes.html), which serves as an endogenous control for RNA extraction. The multiplex reaction was then tested for sensitivity using serial dilutions of *in vitro* transcribed RNA containing the target sequences of all four reactions. The analytical limit of detection (LOD) was determined as the minimal number of target copies detected in all three repeats of the test. As detailed in Fig. S3, the calculated analytical limit of detection for the three viral targets was as follows: 35 copies per reaction for the E-sarbeco reaction, 13.5 copies per reaction for the N_D3L_, and 15.3 copies per reaction for the S_242_ reactions. The analytical LOD for the hRNAse P control reaction was 25 copies per reaction. This sensitivity was accomplished using a reaction that takes less than 70 min, thereby shortening the entire time from sample to answer.

The specificity of the assay was evaluated with a panel of respiratory pathogens, as shown in Fig. S4 and detailed in Table S3. Some of the samples were derived from tissue culture and were not taken directly from clinical samples and were therefore negative for RNase P. However, they all tested positive for their designated pathogen RNA prior to the specificity test being run. Examination of RNA samples from 17 different pathogens showed no cross-detection, including other human coronavirus species (Fig. S4, Table S3).

The multiplex SC-2 VOC assay was then tested using sequenced samples, and the results of the PCR were compared with the sequencing analysis for each sample. As detailed in Table S1, for variant B.1.351, which was significantly less abundant in Israel, 64 samples were examined. For variant B.1.1.7, which was dominant in Israel from February 2021 to June 2021, 200 samples were examined. The comparison showed that all samples in which the defining mutation N_D3L_ was present were correctly identified as B.1.1.7 (Table 2). The quantification cycle (*C_q_*) values of 100 representative B.1.1.7 samples are shown in Table S1. Likewise, 64 samples identified as containing the S_242_ mutation by the multiplex assay, were confirmed to be of variant B.1.351 by the whole-genome sequencing (WGS) analysis (Table 2). These results suggest that the PCR assay can be used with a high degree of certainty to identify these variants (Table 2). For some samples, other mutations were either absent or inconclusive (“N” read in the WGS analysis), but these were less than 10% of the samples in each lineage (Table 2). Notably, in some cases, often when samples with a high viral RNA concentration were tested, weak background signals were observed in the PCR assay (Fig. 2). In some of these samples, a weak signal of the S_242_ reaction was detected in non-B.1.351, such as WT or B.1.1.7 samples, and a weak N_D3L_ reaction was observed in some B.1.351 and WT samples (Fig. 2). In such cases, the E target reaction was used as a reference to evaluate the mutation-specific reaction (either S_242_ or N_D3L_). A *C_q_* difference of up to 3 to 4 cycles was considered a true signal, while a *C_q_* difference of 8 or more was considered a background signal. The *C_q_* difference between the E gene target signal and the background signals, as well as their fluorescence intensity and curvature, clearly indicated that the signal does not reflect the presence of the variant-specific mutations but, rather, reflects a low-affinity binding of the primers to the viral RNA, as shown in Fig. 2. The decision matrix based on the different combinations of the reaction is detailed in Table 3, allowing correct interpretation of such results.

**TABLE 2:** Comparison of the multiplex PCR results and whole-genome sequencing (WGS)^*a*^

Variant mutations	Mutation	B.1.1.7 match (%)	B.1.351 match (%)
	*N:D3L*	*100*	*0*
B.1.1.7	S:H/V69-70 del	100	2
mutations	S:N501Y	100	0
	S:D1118H	100	6
	snp silent NSP2:S36	100	0
	snp silent NSP12b:P430	100	0
	snp silent NSP12b:H604	100	0
	snp silent NSP12b:T903	100	0
	*S:L242:244 del*	*0*	*100*
B.1.351	S:E484K	0	92
mutations	S:K417N	0	84
	snp silent NSP2:T629	0	88
	snp silent ORF8:F120	0	100
	NSP3:K837N	0	92

a The percentage of match between samples identified as B.1.1.7 and B.1.351 by the multiplex assay and the WGS analysis are indicated for each of the mutations below. A total of 200 samples were detected as B.1.1.7, and 64 as B.1.351. The mutations that are targeted in the multiplex assay (N_D3L_ for B.1.1.7 and S_242del_ for B.1.351) are in italics.

**TABLE 3:** Decision matrix of the multiplex reaction results^*a*^

Target/combination	1	2	3	4	5	6
E target	−	−	+	+	+	−
S_242_ target	−	−	−	−/+	ΔE-S_242_ <5	+
N_D3L_ target	−	−	−	ΔE-N_D3L_ >6	−/ΔE-N_D3L_ >7	+
RNAse P target	−	*C_q_* = <35	−/+	−/+	−/+	−/+
Interpretation	Invalid	−for SC-2	SC-2 (+), Non-B.1.1.7, non-B.1.351	B.1.1.7	B.1.351	Retest

a The interpretation for each combination of results is detailed. −, negative; +, positive; ΔE-N_D3L_ > 6, the difference in the *C_q_* values between the E reaction and the N_D3L_ reaction is less than 6 cycles; ΔE-N_D3L_ > 7, the difference in the *C_q_* values between the E reaction and the N_D3L_ reaction is larger than 7 cycles; ΔE-S_242_, the difference in the *C_q_* values between the E reaction and the S_242_ reaction is less than 5 cycles.

### Confirmatory Sanger sequencing of suspected samples.

The first three samples suspected as variant B.1.1.7 (numbered 5824, 6021, and 7075) were negative for the S reaction in the Thermo Fisher SARS-COV-2 test and were subjected to further examination in the Israel Central Virology Laboratory (CVL). Focused Sanger sequencing of the S gene region of samples 5824 and 6021 showed the presence of the 69 to 70 deletion, but the B.1.1.7 variant-associated mutation 144del and N501Y substitution were absent. They both contained, however, a unique substitution, N439K, which is not associated with the B.1.1.7 variant (Fig. S5). Sanger sequencing of the spike gene region from sample 7075 contained the following mutations, all of which are characteristic of the B.1.1.7 variant: 69 to 70 deletion, 144 deletion, N501Y, S982A, and D1118H (Fig. S6). Subsequent complete genome analysis via next-generation sequencing (NGS) of that sample confirmed the presence of all the defining mutations of the B.1.1.7 variant, thereby corroborating the initial results of the Sanger sequencing. This was the first known case of the B.1.1.7 variant in Israel.

Samples suspected as the B.1.351 variant were initially examined by Sanger sequencing of two regions of the spike gene, the 242 to 244 deletion, which is unique to this variant, and the receptor binding domain (RBD; nucleotide positions 22493 to 23218). Out of 20 suspected samples, four contained the following mutations in the spike gene: D215G, the 242 to 244 deletion, K417N, E484K, and N501Y, all associated with the B.1.351 variant (Fig. S6). Subsequent complete genome analysis via NGS confirmed the presence of the B.1.351 defining mutations.

To confirm the accuracy of the multiplex reaction, complete genome sequencing of 122 clinical samples was performed, with complete agreement with the qPCR results. For variant B.1.1.7, over 1,000 samples were examined by both the new qPCR assay and by whole-genome sequencing, with a complete match. These results demonstrate that the new multiplex assay described here can be used as a rapid and reliable approach for primary classification of SC-2 B.1.1.7 and B.1.351 variants.

## DISCUSSION

The emergence of new, more contagious, and potentially antigenically different SC-2 lineages poses an urgent need to adjust rapid detection methods to meet public health-related needs. To meet these needs, we developed a multiplex RT-qPCR assay that can distinguish between three SC-2 lineages. The assay is rapid (∼1 h PCR assay time) and is suitable for high-throughput rapid screening. This is in contrast to Sanger sequencing or NGS, which are more informative but are far more expensive, take significantly more time, and cannot be scaled up easily. Since the beginning of the COVID-19 pandemic, several rapid tests detecting the presence of SC-2 RNA or proteins were implemented in wide-scale testing (11). However, the capacity to distinguish between different lineages in less than 2 h is currently possible only using qPCR.

SC-2 variant B.1.1.7 contains numerous synonymous and nonsynonymous mutations, of which the spike gene mutations 69-70del, N501Y, and P681H received most attention due to their potential effect on virus infectivity (12, 13). For diagnostic purposes, however, the N501Y mutation is not variant-specific, as it was identified in several variants other than variant B.1.1.7, such as B.1.351 and the P.1 variant (14). The D3L substitution in the N gene used in our assay is specific to variant B.1.1.7 and was not reported in other major SC-2 lineages. Although this mutation can occur independently of other characteristic mutations, such as N501Y, its presence strongly suggests that the examined sample is the B.1.1.7 variant. Likewise, the reaction that identifies the variant B.1.351 targets mutations that are strongly associated with this variant—D215G and the triple deletion of amino acids 242 to 244 (Fig. S4). The combinations of these two reactions therefore provide a reliable tool to identify each of these two variants with high confidence. The agreement between the multiplex PCR results and the WGS analysis performed on over 250 samples suggested that this assay is a reliable tool to rapidly classify suspected samples as B.1.1.7, B.1.351, or neither. It also allows us to determine the integrity of the sampling and extraction procedure using the control hRNAse P reaction.

In order to increase the range of the new assay, the SC-2 inclusive reaction targeting the E gene (10) was combined, thereby enabling detection of the viral RNA independently of the strain examined.

A few commercial kits partially address the detection of these SC-2 variants, but they target general mutations, such as N501Y and E484K in the spike protein, and not variant-specific mutations, such as the N protein D3L substitution or the spike protein 242 to 244 deletion (https://www.seegene.com/assays/rp, http://www.kogene.co.kr/eng/sub/product/covid-19.asp).

The emergence of novel SC-2 variants with increased infectivity and increased resistance to current vaccines may significantly impair global large-scale detection and vaccination efforts (15). Moreover, it has been shown that different variants having some identical mutations in the spike coding sequence still have different infectivity and vaccine resistance characteristics due to their different sets of additional mutations (5, 9; M. Mandelboim, unpublished data). As a result, the pressing need to improve detection methods accordingly requires constant adjustments. Such diagnostic tests should not only detect the presence of the viral RNA with high specificity and sensitivity, but also provide information on variant identity. An additional consideration is the relatively high cost of commercial kits and the need to perform complex interpretation of the results to determine the possible sample identity. The execution and analysis of the assay described here are simple and relatively inexpensive compared with current commercial kits. Implementation of molecular assays such as our multiplex qPCR assay will improve SC-2 diagnosis and contribute to the ongoing efforts to contain the COVID-19 pandemic.

## MATERIALS AND METHODS

### Design of VOC-specific qPCRs.

Analysis of SC-2 sequences and primer simulations were performed using the Geneious software package and the NCBI BLAST analysis tools (https://blast.ncbi.nlm.nih.gov/). SC-2 sequences were obtained from the GISAID initiative website (https://www.gisaid.org/) and analyzed using the Geneious software. All primer and probe sequences are detailed in Table 1.

### Processing of SC-2 clinical samples.

Nasopharyngeal swab samples suspected to contain SC-2 in viral transport medium (VTM) were inactivated by heating at 70°C for 30 min or, if intended to be used for culturing, were inactivated by addition of 200 μl lysis buffer (Zymo Research, to 200 μl VTM. Total RNA extraction was performed with either the Roche MagNA Pure 96 system or the PSS MagLEAD instrument. The eluted RNA was stored at −80°C for further use or used immediately thereafter for the PCR test.

### Design and synthesis of *in vitro* transcribed standard RNA segments.

In order to establish the analytical limit of detection (LOD) and obtain standard controls for WT and mutant target sequences, genomic regions, including the E, S_242_, S_RBD_, N, and RNASE P, were amplified using primers that contain the T7 promoter minimal sequence (Table 4) with the MyTaq one-step RT-PCR kit. The resulting PCR products were transcribed *in vitro* to RNA using the T7 MEGAscript kit according to the manufacturer’s instructions (Thermo Fisher). The *in vitro* transcribed RNA was purified, and its concentration was determined using a Nanodrop spectrophotometer and stored at −80°C.

**TABLE 4:** Cloning reaction design^*a*^

Mutations within product	Fwd primer 5′→3′	Rev primer 5′→3′	Product size
Spike 69-70Del, 144Del, D215G, 242Del	Cov19 21906 TTAGATTCGAAGACCCAGTCCCTAC	Cov19 22475 GTTTCTGAGAGAGGGTCAAGTGCAC	570 bp
Spike K417N, E484K, N501Y	CoV19 22509 GGAATCTATCAAACTTCTAACTTTAGAG	CoV19 23233 AGAACACCTGTGCCTGTTAAACCATTG	725 bp
Spike S982A, D1118H	Cov19 24185 GATTGCTCAATACACTTCTGCAC	CoV19 24716 TGAGGGAAGGACATAAGATGATAG	555 bp
N D3L	nCoV 28225 F GAAGACTTTTTAGAGTATCATGAC	COV19 29297 GGATCTTTGTCATCCAATTTGATG	1,090 bp

a In the left column are the gene name and the amino acid position of mutations within the amplified sequence. The forward (Fwd) and reverse (Rev) primer sequences are detailed in the middle columns. The expected PCR product size is shown in the right column. All primers were designed in this study.

### RT-qPCR.

RT-qPCR mix was prepared with the Meridian (formerly Bioline) SensiFast one-step mix. Initial optimization was performed by setting an annealing temperature gradient. Following optimization of the reaction conditions, the final mix concentration was determined. The reaction mix was assembled as follows: SensiFast Probe Lo-ROX one-step, 12.5 μl; E Sarbeco-F1b, 400 nM; E-Sarbeco-R, 400 nM; E-Sarbeco probe, 200 nM; 21257VOC forward (Fwd), 750 nM; CDC N1 reverse (Rev), 750 nM; CDC N1 probe, 300 nM; 22201B SA Fwd, 600 nM; 22238 SA Rev, 800 nM; 22230 probe, 300 nM; RNASE P Fwd, 300 nM; RNASE P Rev, 300 nM; RNASE P probe, 200 nM; RT enzyme, 0.2 μl; RNase inhibitor, 0.2 μl; double-distilled water (ddH_2_O) to a final volume of 1 μl.

The amplification was performed in a Bio-Rad CFX96 thermal cycler using the following conditions: (i) 45°C for 10′, (ii) 95°C for 2′, 20″, 45× ([ii] 95°C for 4″, [d] 62.2°C for 28″). Fluorescence was recorded at each cycle during the annealing and extension step (62.2°C for 28″). The reaction data were analyzed using Bio-Rad CFX Maestro software.

Each PCR run included at least one positive control of SC-2 RNA, and one negative control of H_2_O, as is the standard for any PCR test performed in the CVL.

### Sanger sequencing of suspected samples.

In order to identify mutations of the variants of interest in suspected samples, several rapid sequencing reactions of the Spike gene were designed and implemented. This enabled the first identification of the B.1.1.7-related mutations 69-70Del, 144Del, N501Y, S982A, and D1118H and B.1.351-related mutations D215G, 242Del K417N, N501Y, and E484K. Primer sets for the rapid sequencing reactions of the spike and nucleocapsid (N) genes are detailed in Table 4.

Endpoint PCRs were performed with a MyTaq one-step RT-PCR kit (Meridian) according to the manufacturer’s instructions. The resulting PCR products were analyzed using agarose gel electrophoresis and sequenced using the ABI 3500 Bioanalyzer.

### Next-generation whole-genome sequencing of clinical samples.

A COVID-seq kit was used for library preparation as per the manufacturer’s instructions (Illumina). Library validation and mean fragment size were determined using TapeStation 4200 with a DNA HS D1000 kit (Agilent). Libraries were pooled, denatured, and diluted to 10 pM and sequenced on a NovaSeq instrument (Illumina).

### Bioinformatic and phylogenetic analysis.

Fastq files underwent quality control using FastQC (www.bioinformatics.babraham.ac.uk/projects/fastqc/) and MultiQC (16), and low-quality sequences were filtered using Trimmomatic (PMID: 24695404). Mapping to the SARS-CoV-2 genome (GenBank accession number NC_045512.2) was performed with the Burrows-Wheeler Aligner MEM algorithm (BWA-MEM) (PMID: 19451168). The SAMtools suite (PMID: 19505943) was used to filter unmapped reads, and sort and index bam files. Consensus fasta sequences were constructed for each sample using iVar (https://andersen-lab.github.io/ivar/html/manualpage.html), with Ns inserted in positions with a sequencing depth lower than 5. Sequences were aligned with the SARS-CoV-2 reference sequence (GenBank accession number NC_045512.2) with MAFFT (17), and mutation analysis was done with a custom R code using the Bioconductor package seqinr (https://cran.r-project.org/web/packages/seqinr/).
