# Supplementary material for: Rapid and High-Throughput Reverse Transcriptase Quantitative PCR (RT-qPCR) Assay for Identification and Differentiation between SARS-CoV-2 Variants B.1.1.7 and B.1.351
Source: Microbiol Spectr. 2021 Oct 6;9(2):e00506-21. doi: 10.1128/Spectrum.00506-21 (PMC8510166; doi:10.1128/Spectrum.00506-21)
Supplement: SUPPLEMENTAL FILE 1 — Supplemental material. Download SPECTRUM00506-21_Supp_1_seq6.pdf, PDF file, 2.5 MB [file spectrum00506-21_supp_1_seq6.pdf]

Supplementary Material File

Rapid And high throughput RT-qPCR assay for identification and differentiation between SARS-CoV-2 variants B.1.1.7 and B.1.351.

Oran Erster[1§], Ella Mendelson[1,2], Virginia Levy[1], Areej Kabat[1], Batya Mannasse[1], Hadar Asraf[1], Roberto Azar[1], Yaniv Ali[1], Rachel Shirazi[1], Efrat Bucris[1], Dana Bar-Ilan[1], Orna Mor[1,2], Michal Mandelboim[1,2], Danit Sofer[1], Shai Fleishon[1], Neta S Zuckerman[1]

[1]Central Virology Laboratory, Public Health Services, Ministry of Health, Chaim Sheba Medical Center, Ramat Gan, Israel.

[2]School of Public Health, Sackler Faculty of Medicine, Tel-Aviv University, Tel-Aviv, Israel.

[§]Corresponding author contact information: oran.erster@sheba.health.gov.il

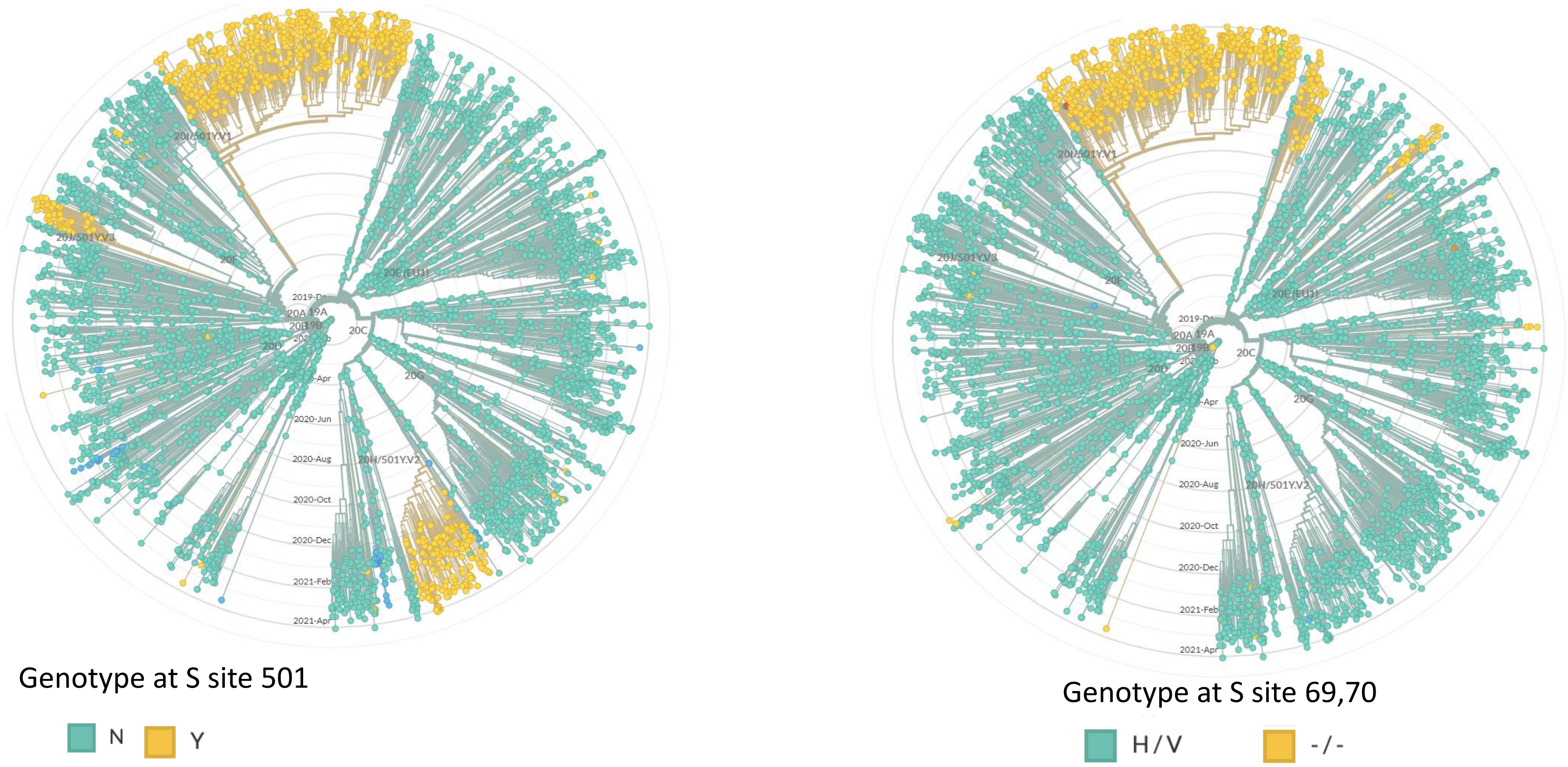

<u>Supplemental Figure S1</u>. Global phylogenetic alignment of SARS-COV-2 lineages, showing the Prevalence of N501Y (left) and 69-70del (right) mutations. The WT sequence (N for the 501 plot and H/V for the 69-70 plot) is colored in grey. The mutation (Y for the 501 plot and -/- for the 69-70 plot) is highlighted in yellow. Lineage annotations: 20I/501Y.V1 – B.1.1.7, 501.V2, 20H/501Y.V2 -B.1.351, 20J/501Y.V3 – P1 . Plots were generated using the Nextstrain website (https://nextstrain.org/ncov/).

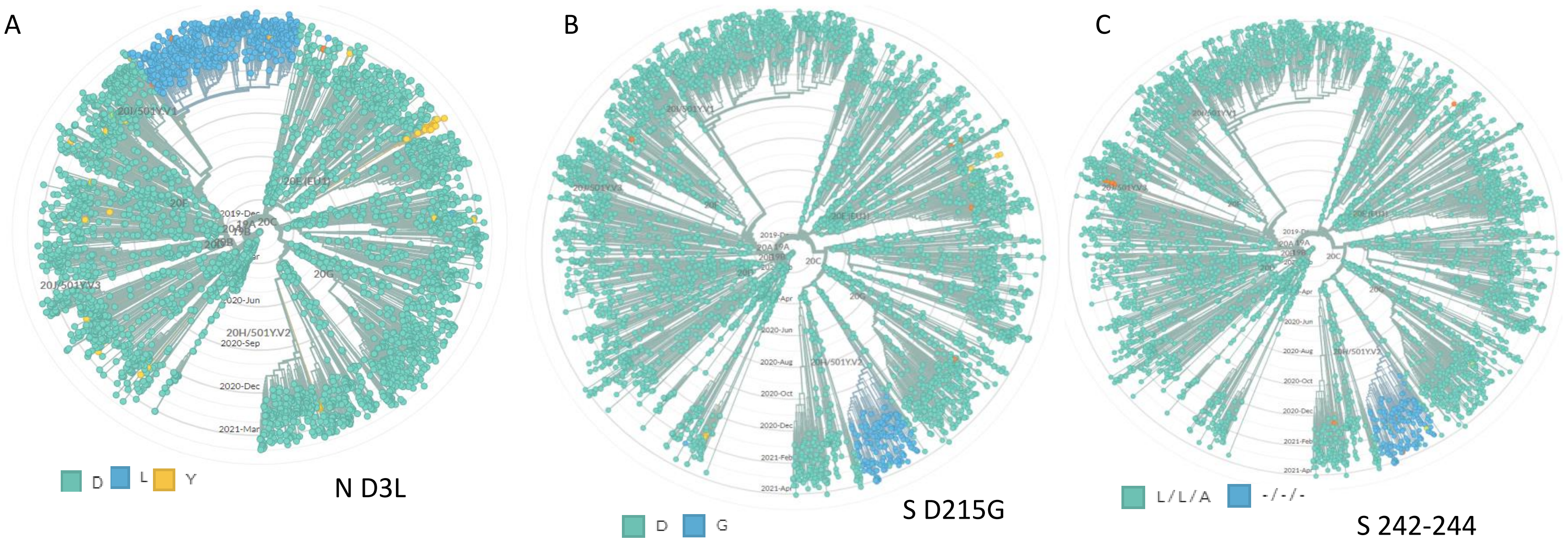

<u>Supplemental Figure S2</u>. **Global phylogenetic alignment of SARS-COV-2 lineages, showing the Prevalence of N (nucleocapsid), Spike (S) D215G and S 242-244del mutations.** (A) Global alignment of SC-2 showing the different mutations in position 3 of the N gene. The L substitution is colored in purple. (B) Global alignment of showing the presence of the S D215G mutation, highlighted in purple. (C) Global alignment of showing the presence of the S 242-244 deletion. The WT sequence (D for the N D3L plot, D for the D215G plot and L/L/A for the 242-244 plot) is colored in grey. Lineage annotations: 20I/501Y.V1 – B.1.1.7, 501.V2, 20H/501Y.V2 -B.1.351, 20J/501Y.V3 – P1. Plots were generated using the Nextstrain website (https://nextstrain.org/ncov/).

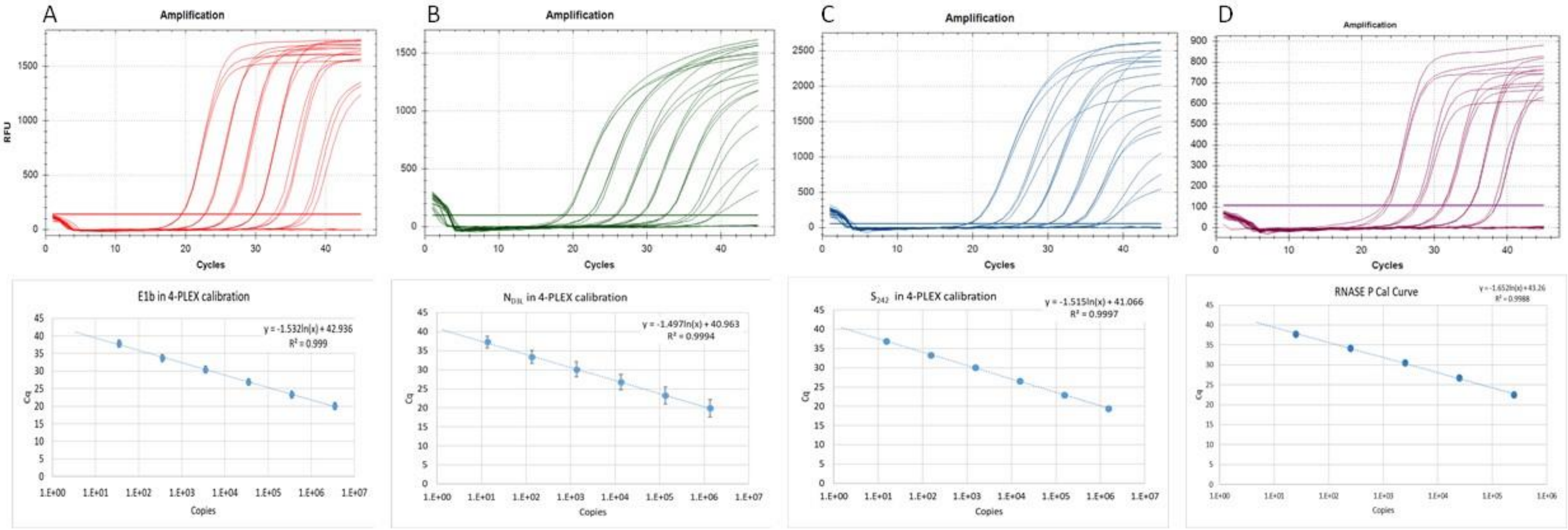

<u>Supplementary Figure S3.</u> Standard calibration of the multiplex assay reactions. *In-vitro* transcribed RNA molecules corresponding to the target sequences were serially diluted and tested in triplicates. The average Cq values for each concentration were plotted against the calculated RNA copies. The resulting calibration formula and the R^2 value of the logarithmic regression line appear in the inset of each graph. For each reaction, the raw amplification curves (Top) and logarithmic regression graph (Bottom) are shown. (A) CoV19 E target reaction (B) CoV19 N_{D3L} reaction (C) CoV19 S_{242} reaction (D) human RNAseP reaction.

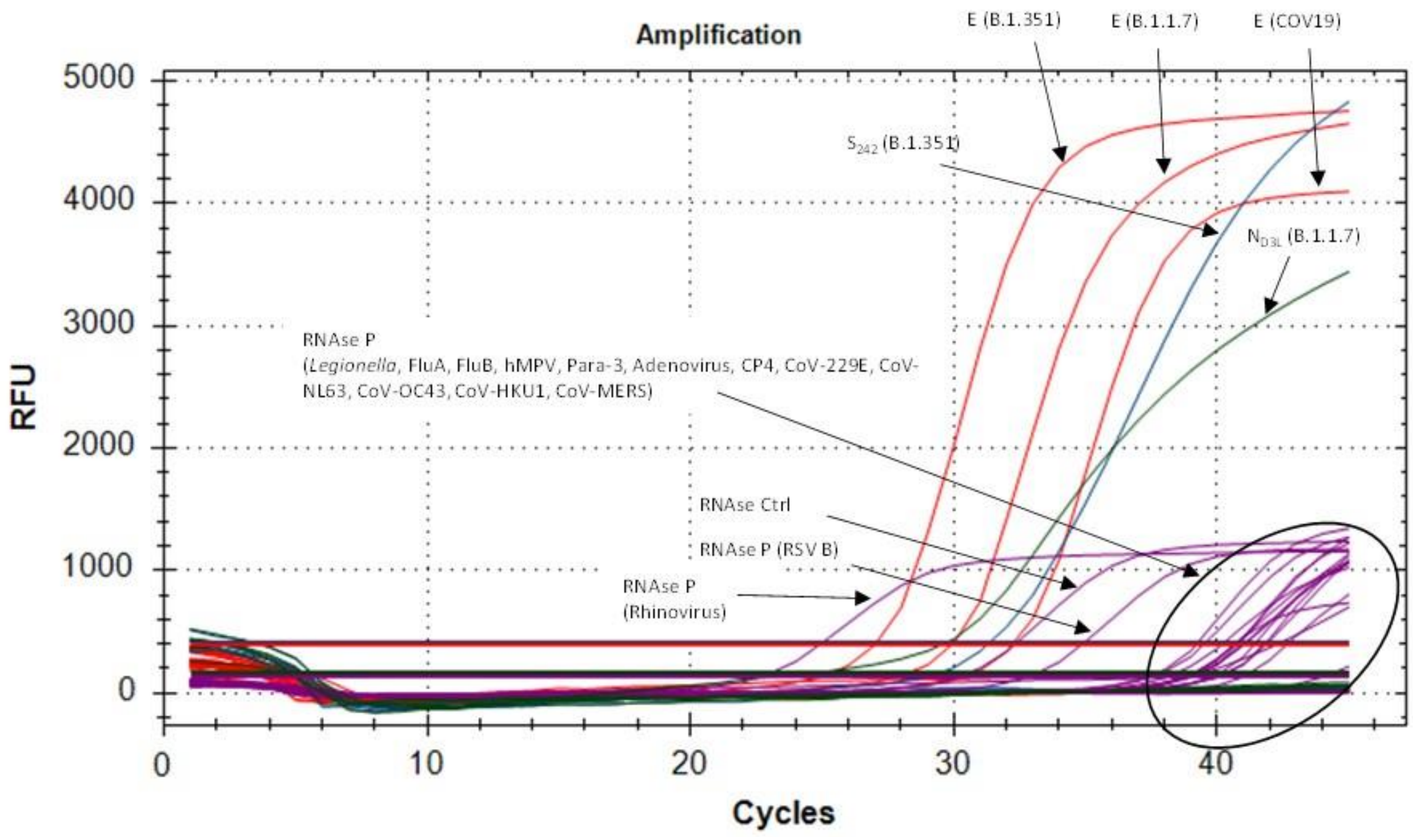

<u>Supplementary Figure S4.</u> Amplification curves of the multiplex assay specificity test. Amplification curves of different respiratory pathogen samples in the multiplex assay. For each curve, the target reaction is denoted and the sample name is in parenthesis. Pathogen name abbreviations: Cp-4: *Chlamydia pneumoniae*, SW: Swine flu, CoV: Coronavirus, MP-3: *Mycoplasma pneumonia*, hMPV - Human metapneumovirus, RSV: Respiratory syncytial virus, Para-3: Parainfluenza virus 3, Legionella: *Legionella ssp.*

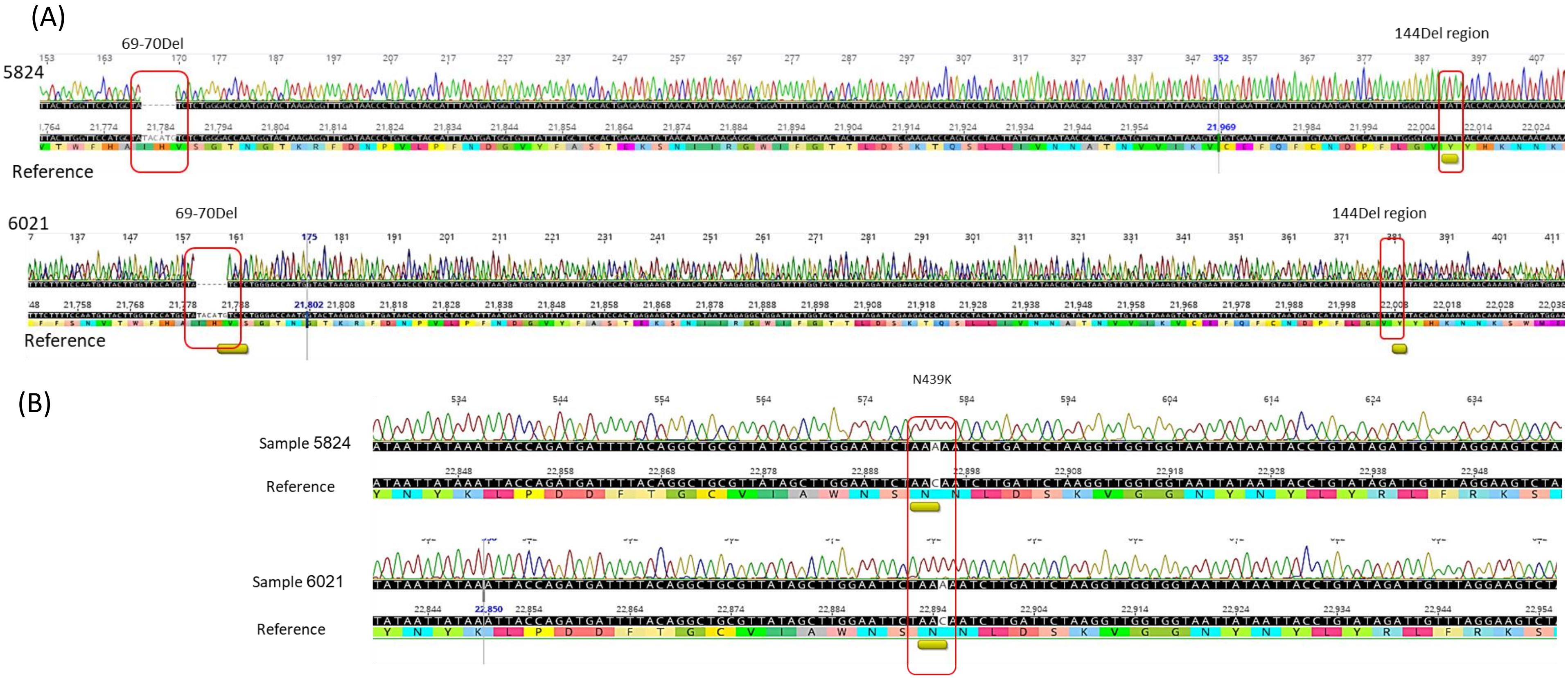

<u>Supplemental Figure S5</u>. Alignment of lineage B.1.1.7 suspected samples with reference sequence NC_045512. In samples 5824 and 6021, The deletion of 69-70 was evident, but the 144 deletion was absent (A). Both sample did not contain any characteristic mutations of lineage B.1.1.7 in the RBD, but did contained the N439K mutation, which is not a defining mutation of variant B.1.1.7 (B). Mutations are marked with a rectangle and an underline under the sequence. Reference sequence: accession NC_045512.

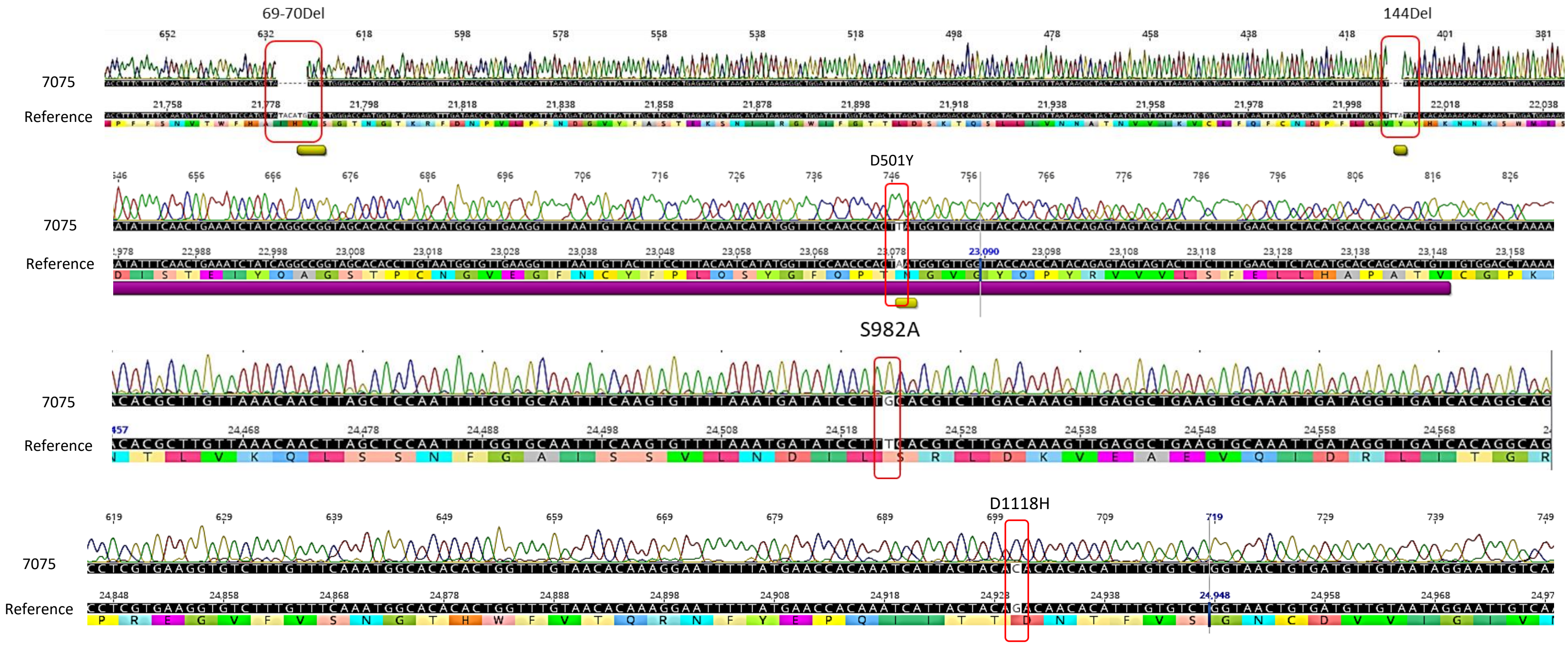

<u>Supplemental Figure S6.</u> Sequencing of the Spike gene N-terminal and RBD regions of sample 7075. The following Variant B.1.1.7- associated mutations were detected: 69-70 deletion, 144 deletion, D501Y, S982A and D1118H. Mutations are marked with a rectangle and an underline under the sequence. Reference sequence: accession NC_045512. Violet underline in the second alignment marks the RBD region.

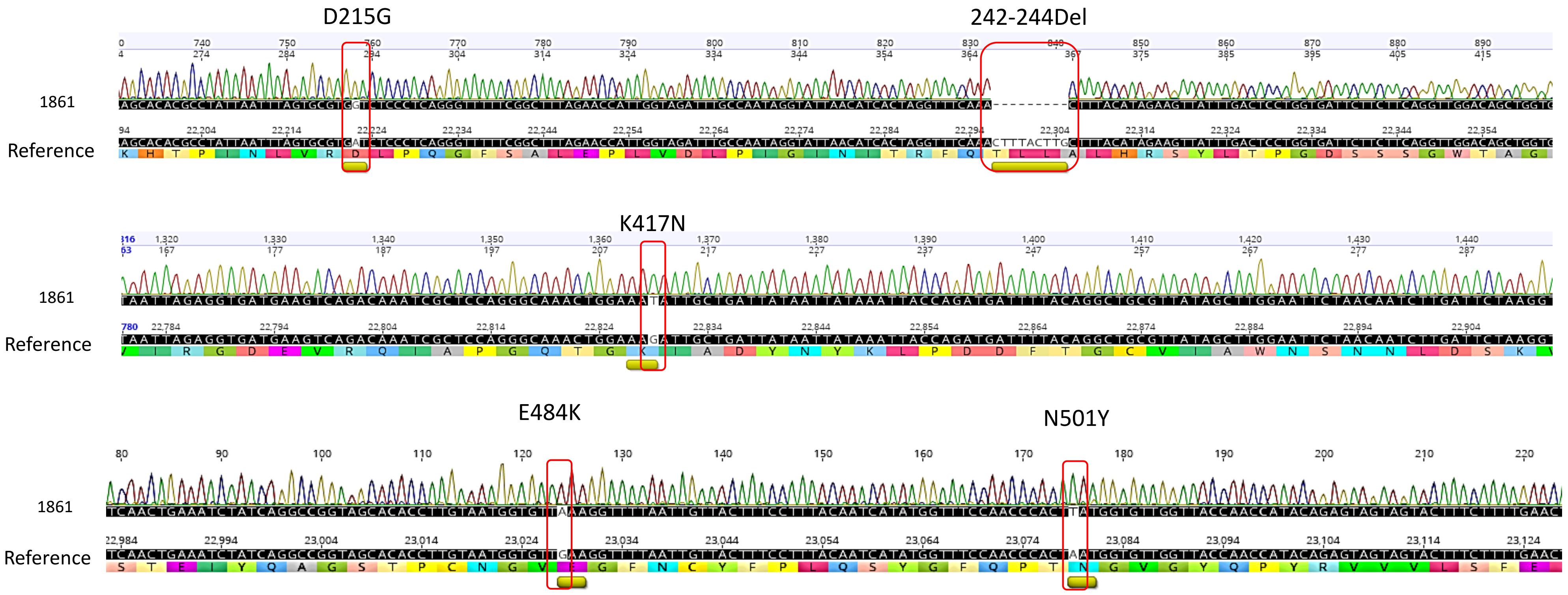

Supplementary Figure S7. Alignment of representative Spike gene N-terminal and RBD regions sequences of variant B.1.351 - suspected sample. The following mutations associated with variant B.1.351 were detected: D215G, 242-244del, K417N, E484K and N501Y. Mutations are marked with a rectangle and an underline under the sequence. Reference sequence: accession NC_045512.

Supplementary Table S1. Comparison of whole genome sequencing analysis and the multiplex PCR results of variant B.1.1.7 samples. The Cq value obtained for each sample in the E reaction (E CVL) and the N_{D3L} reaction (N_{D3L} CVL) are indicated, together with the Pangolin clade assigned following whole genome sequencing analysis.

Sample	E-CVL	N_{D3L} CVL	WGS analysis	Sample	E-CVL	N_{D3L} CVL	WGS analysis
6614	24.04	23.47	B.1.1.7	6842	20.71	18.40	B.1.1.7
6617	31.06	29.72	B.1.1.7	6843	24.39	21.50	B.1.1.7
6618	25.81	24.05	B.1.1.7	6844	29.04	26.48	B.1.1.7
6655	13.50	12.11	B.1.1.7	6845	17.17	13.96	B.1.1.7
6656	17.91	17.54	B.1.1.7	6846	22.56	20.28	B.1.1.7
6658	17.25	15.35	B.1.1.7	6847	24.17	22.55	B.1.1.7
6659	24.68	22.67	B.1.1.7	6848	26.17	23.33	B.1.1.7
6660	30.23	28.58	B.1.1.7	6849	22.18	20.11	B.1.1.7
6661	30.02	28.24	B.1.1.7	6850	26.78	24.04	B.1.1.7
6662	29.54	23.75	B.1.1.7	6851	26.11	24.59	B.1.1.7
6663	28.12	24.78	B.1.1.7	6852	23.04	20.08	B.1.1.7
6664	22.85	19.86	B.1.1.7	6853	21.03	19.03	B.1.1.7
6666	23.56	20.42	B.1.1.7	6854	25.62	24.27	B.1.1.7
6667	22.73	19.82	B.1.1.7	6855	23.77	21.82	B.1.1.7
6668	21.41	20.20	B.1.1.7	6856	21.28	18.79	B.1.1.7
6669	25.81	22.14	B.1.1.7	6857	24.48	22.16	B.1.1.7
6670	28.72	25.46	B.1.1.7	6858	21.95	18.81	B.1.1.7
6671	27.82	22.98	B.1.1.7	6859	22.65	20.70	B.1.1.7
6672	24.95	19.47	B.1.1.7	6860	27.00	25.10	B.1.1.7
6807	21.39	18.85	B.1.1.7	6861	25.05	23.07	B.1.1.7
6808	25.23	23.52	B.1.1.7	6862	25.67	24.27	B.1.1.7
6809	25.12	22.07	B.1.1.7	6863	25.60	24.17	B.1.1.7
6810	20.44	17.50	B.1.1.7	6864	26.44	25.07	B.1.1.7
6811	22.64	20.34	B.1.1.7	6865	23.24	20.30	B.1.1.7
6813	24.19	22.42	B.1.1.7	6866	30.45	30.22	B.1.1.7
6814	30.75	29.04	B.1.1.7	6867	21.61	19.75	B.1.1.7
6815	25.50	22.99	B.1.1.7	6869	31.21	26.82	B.1.1.7
6816	25.33	22.97	B.1.1.7	6872	21.25	18.11	B.1.1.7
6817	26.99	26.54	B.1.1.7	6873	24.16	18.18	B.1.1.7
6818	19.33	19.83	B.1.1.7	6874	24.06	19.63	B.1.1.7
6819	22.32	21.44	B.1.1.7	6875	25.32	23.58	B.1.1.7
6820	27.08	25.54	B.1.1.7	6876	29.87	26.02	B.1.1.7
6821	22.14	19.42	B.1.1.7	6883	20.24	19.33	B.1.1.7
6823	26.75	25.13	B.1.1.7	6889	22.29	19.90	B.1.1.7
6825	25.28	25.39	B.1.1.7	6892	23.34	20.69	B.1.1.7
6826	21.84	19.78	B.1.1.7	6893	29.69	28.24	B.1.1.7
6827	25.43	23.29	B.1.1.7	6895	25.84	23.01	B.1.1.7
6828	26.54	24.72	B.1.1.7	6896	30.21	29.65	B.1.1.7
6829	22.19	20.25	B.1.1.7	6897	29.60	28.09	B.1.1.7
6830	25.28	23.53	B.1.1.7	6923	17.24	15.29	B.1.1.7
6831	26.98	27.81	B.1.1.7	6924	22.33	21.89	B.1.1.7
6832	22.39	19.59	B.1.1.7	6925	22.08	20.58	B.1.1.7
6833	22.21	19.31	B.1.1.7	6927	22.02	20.65	B.1.1.7
6834	22.03	19.37	B.1.1.7	6928	19.74	18.14	B.1.1.7
6835	29.05	26.92	B.1.1.7	6929	25.69	24.25	B.1.1.7
6836	20.41	17.48	B.1.1.7	6930	19.73	17.89	B.1.1.7
6837	21.36	18.18	B.1.1.7	6931	27.85	26.89	B.1.1.7
6838	25.80	23.54	B.1.1.7	6932	21.67	20.45	B.1.1.7
6839	25.42	22.43	B.1.1.7	6933	22.99	21.68	B.1.1.7
6840	24.00	21.24	B.1.1.7	6934	24.65	22.68	B.1.1.7

Supplementary Table S2. Comparison of whole genome sequencing analysis and the multiplex PCR results of variant B.1.351 samples. The Cq value obtained for each sample in the E reaction (E CVL) and the $S_{242-244}$ reaction (S_{242} CVL) are indicated, together with the Pangolin clade assigned following whole genome sequencing analysis.

Sample	E-CVL	S_{242} CVL	WGS analysis
5574	28.28	30.15	B.1.351
5878	23.39	25.17	B.1.351
5904	24.14	25.67	B.1.351
5923	26.91	28.73	B.1.351
6289	22.25	22.14	B.1.351
6360	23.79	34.86	B.1.351
6361	30.85	31.46	B.1.351
6363	31.44	29.30	B.1.351
6364	27.83	29.44	B.1.351
6366	33.43	25.09	B.1.351
6368	30.33	22.18	B.1.351
6370	32.04	31.12	B.1.351
6374	25.37	20.82	B.1.351
6382	18.90	27.27	B.1.351
6461	19.98	23.90	B.1.351
6463	17.66	15.75	B.1.351
6464	26.35	24.74	B.1.351
6466	25.90	32.93	B.1.351
6467	24.02	29.73	B.1.351
6468	30.62	27.74	B.1.351
6470	16.30	19.58	B.1.351
6471	22.50	27.87	B.1.351
6472	15.29	20.10	B.1.351
6474	15.39	21.31	B.1.351
6475	18.92	23.00	B.1.351
6491	26.13	28.21	B.1.351
6593	21.72	25.07	B.1.351
6595	14.92	17.53	B.1.351
6596	20.39	23.38	B.1.351
6603	23.79	26.09	B.1.351
6604	22.58	24.02	B.1.351
6606	27.57	29.94	B.1.351

Sample	E-CVL	S_{242} CVL	WGS analysis
6607	20.46	23.01	B.1.351
6608	19.99	21.86	B.1.351
6609	22.99	26.30	B.1.351
6610	23.83	25.00	B.1.351
6612	29.87	31.12	B.1.351
6613	29.37	30.82	B.1.351
6615	22.42	21.22	B.1.351
6616	27.83	29.71	B.1.351
6619	19.07	21.87	B.1.351
6623	27.94	30.12	B.1.351
6627	22.29	24.37	B.1.351
6629	26.42	29.35	B.1.351
6642	21.20	23.03	B.1.351
6871	22.13	26.68	B.1.351
6877	20.07	24.42	B.1.351
6891	24.02	28.25	B.1.351
7065	21.17	23.93	B.1.351
7480	21.40	25.15	B.1.351
7488	19.62	23.89	B.1.351
7494	27.52	31.62	B.1.351
7495	28.84	33.22	B.1.351
7496	21.50	24.84	B.1.351
7588	24.14	28.63	B.1.351
7598	31.05	43.32	B.1.351
7713	19.90	23.64	B.1.351
7714	25.37	30.06	B.1.351
7715	26.19	31.77	B.1.351
7732	20.59	24.44	B.1.351
7802	19.36	28.60	B.1.351
8163	30.32	32.71	B.1.351
8230	27.07	31.00	B.1.351
8238	28.61	33.04	B.1.351

<u>**Supplementary Table S3**</u>. **Specificity test of the new multiplex assay.** The assay was tested with nucleic acid from the pathogens listed below. The Cq values for each reaction in the assay is shown. N/A – No amplification. Cp-4: *Chlamydia pneumoniae*, SW: Swine flu, CoV: Coronavirus, MP-3: *Mycoplasma pneumonia*, HMPV - Human metapneumovirus, RSV: Respiratory syncytial virus, PARA-3: Parainfluenza virus 3.

Sample	E CVL	N_{D3L}	S_{242}	RNAse P
SC-2 WT	32.18	N/A	N/A	38.73
B.1.351	26.98	N/A	30.73	N/A
B.1.1.7	29.88	25.04	N/A	38.95
CP-4	N/A	N/A	N/A	38.82
Flu A	N/A	N/A	N/A	38.09
Flu B	N/A	N/A	N/A	38.85
SW H1	N/A	N/A	N/A	44.05
CoV HKU-1	N/A	N/A	N/A	41.03
CoV NL-63	N/A	N/A	N/A	38.98
CoV OC43	N/A	N/A	N/A	39.85
MERS CoV	N/A	N/A	N/A	39.10
CoV 229E	N/A	N/A	N/A	43.88
Legionella spp.	N/A	N/A	N/A	40.00
MP-3	N/A	N/A	N/A	N/A
hMPV	N/A	N/A	N/A	37.46
Rhinovirus	N/A	N/A	N/A	22.62
RSV A	N/A	N/A	N/A	N/A
RSV B	N/A	N/A	N/A	32.77
Adenovirus	N/A	N/A	N/A	37.29
PARA-3	N/A	N/A	N/A	38.73
